# Modified Baby Milk—Bioelements Composition and Toxic Elements Contamination

**DOI:** 10.3390/molecules26144184

**Published:** 2021-07-09

**Authors:** Agnieszka Maruszewska, Wojciech Żwierełło, Marta Skórka-Majewicz, Irena Baranowska-Bosiacka, Agata Wszołek, Katarzyna Janda, Daria Kulis, Patrycja Kapczuk, Dariusz Chlubek, Izabela Gutowska

**Affiliations:** 1 Institute of Biology, University of Szczecin, Felczaka 3c St., 71-412 Szczecin, Poland; anieszka.maruszewska@usz.edu.pl (A.M.); agata.wszolek@usz.edu.pl (A.W.); 2 Molecular Biology and Biotechnology Center, Institute of Biology, University of Szczecin, Wąska 13 St., 71-415 Szczecin, Poland; 3 Department of Medical Chemistry, Pomeranian Medical University, Powstańców Wlkp. 71 St., 70-111 Szczecin, Poland; wojciech.zwierello@gmail.com (W.Ż.); marta_skorka@o2.pl (M.S.-M.); misiu1805@op.pl (D.K.); 4 Department of Biochemistry, Pomeranian Medical University, Powstańców Wlkp. 72 St., 70-111 Szczecin, Poland; ika@pum.edu.pl (I.B.-B.); Patrycja.kapczuk@pum.edu.pl (P.K.); dchlubek@pum.edu.pl (D.C.); 5 Department of Human Nutrition and Metabolomic, Pomeranian Medical University, Broniewskiego 24 St., 71-460 Szczecin, Poland; Katarzyna.Janda@pum.edu.pl

**Keywords:** modified milk, nutritional standards, toxic elements, food contamination, infants feeding

## Abstract

Breast milk has the most suitable composition for the proper development in the first year of a child’s life. However, it is often replaced with artificial milk. The aim of the study was to analyze the composition of essential elements: Na, K, Ca, P, Mg, Fe, Zn, Cu, and Mn as well as toxic elements: Ni, Pb, Sr, Li, and In in 18 formulas available in Poland. The daily supply was also estimated. The study was performed by Inductively Coupled Plasma Optical Emission Spectrometry method. The results showed the presence of all essential elements tested, but the content of P and Mn significantly differed from the concentrations declared. Such discrepancies can have significant impact on the daily dose of the bioelements taken. However, the content of elements was within the reference standards established by the EU Directive with exception of P, the amount of which exceeded the norms 5.23–18.80-times. Daily supply of P in tested milk as well as Fe and Mn provided with first and hypoallergenic formula exceeded the adequate intake. Analysis revealed the contamination with harmful elements—Pb, Sr, Li, and In were detected in almost all products. The study confirms the data concerning some discrepancies in composition and the contamination of food and may provide information on the feeding quality of children and estimation of health risk associated with exposure to toxic elements.

## 1. Introduction

Although breast milk is the recommended nutritional product for newborns and infants, modified milk feeding is a common feeding choice that replaces breastfeeding partially or completely. Sometimes the decision to feed a baby with modified milk is a necessity due to medical procedures, diseases, allergies, a child’s specific nutritional needs, or social problems [1,2,3].

Artificial milk for young children is food for particular nutritional purposes. The production of this type of food is subject to strict legal regulations, both international and country-specific. However, the regulations regarding the qualitative and quantitative composition cover only a strictly defined range of substances and the data declared by manufacturers may not fully reflect the actual composition of the product. Moreover, in the literature on the subject, there is little data on the safety of using modified milk in terms of toxic contaminations [4,5].

Breastfeeding is basically recognized as the best form of infant nutrition from a medical and nutritional point of view. The ingredients of human milk, with a properly balanced mother’s diet, provide a baby with the adequate amounts of nutrients and minerals. Moreover, they also ensure immunologic and growth factors necessary for proper development [6,7,8,9].

Infant milk, both natural and artificial (cow milk-, soy-, or protein hydrolysates-based), should meet the nutritional needs of children not only in terms of energy and building components, but also macro- and microelements [10].

An important problem is the contamination of food products. Chemical contaminants in the food product can come from raw material, water, production facilities, or packaging. Another important problem are the differences between the actual and declared content of ingredients. Both such cases constitute a significant nutritional problem [11,12]. Contamination with harmful elements disrupts the functioning and development of the body. In the literature on the subject, there are few data on the contamination of modified baby milk with toxic elements such as: lead, cadmium, arsenic, nickel, aluminum, vanadium, antimony, and thallium [13,14,15,16,17], the toxicity of which for humans is well described [18]. The data obtained indicate a significant variation in the quantitative composition. In many cases, toxic trace elements were present at acceptable levels. On the other hand, there have been cases where the contamination with these elements in analyzed baby milk, significantly exceeds the permissible standards.

The discrepancies in the nutritional composition may affect the daily amount of food ingredients, different from the recommended standards [19,20,21]. The discrepancy between the declared content of the desired ingredients and the actual state very often results from “overages”, when ingredients are added in larger amounts as their amount decreases during storage [22]. Nevertheless, while accepted, these practices may contribute to physiological and metabolic disturbances in the body.

Nutritional errors, infant feeding methods and nutritional products choices are essential for the proper development in further stages of individual development. Furthermore, the accuracy and high quality of baby food production processes also play an important role in this regard [23,24,25,26]. The results presented in the article expand the current state of knowledge in the research on food safety for children.

## 2. Results

### 2.1. Bioelements Content in Dry Powder of Analyzed Milk

**Figure 1:**
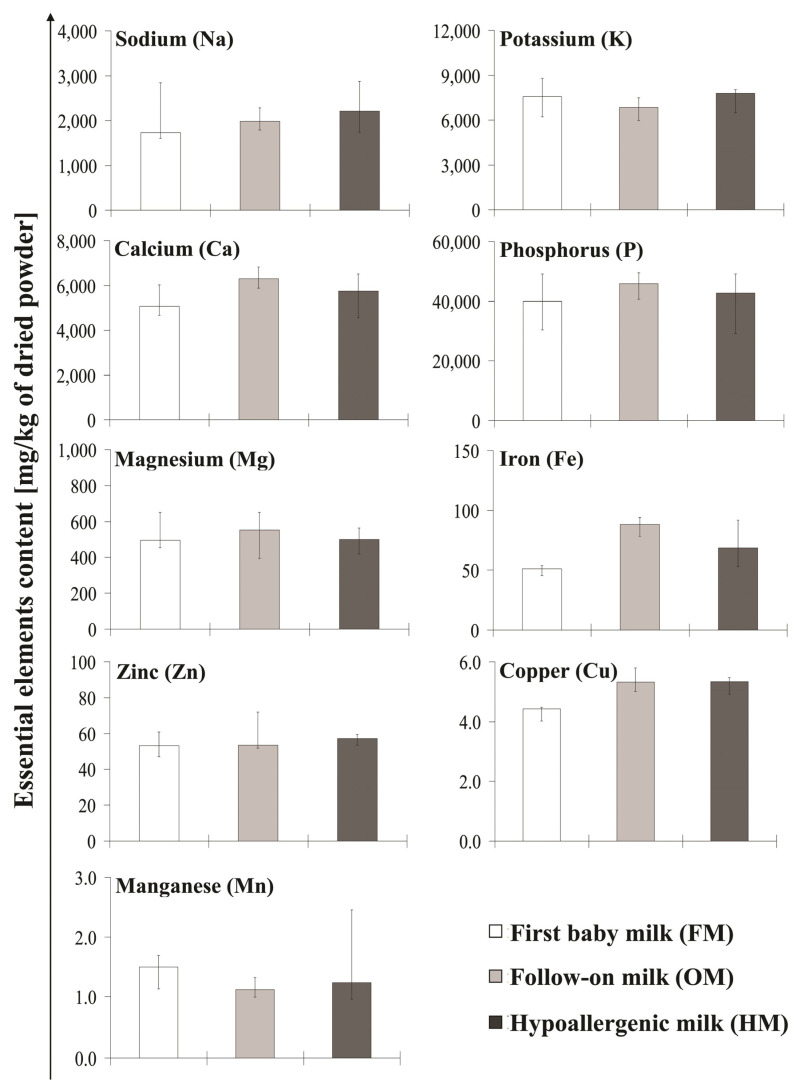
Concentration of the essential elements contained in analyzed baby milk. The values of sodium (Na), potassium (K), calcium (Ca), phosphorus (P), magnesium (Mg), iron (Fe), zinc (Zn), copper (Cu), and manganese (Mn) content in analyzed first baby milk (FM), follow-on milk (OM), and hypoallergenic milk (HM). The data are presented as median (interquartile range) values of six determinations.

The concentrations of macro-, and microelements in investigated first baby milk (FM), follow-on milk (OM), and hypoallergenic milk (HM) are presented in Table 1. The concentration values are given as a median value (with minimum and maximum level) for each analyzed product group. The contents of the essential elements in individual milk groups are shown in Figure 1.

Essential elements, the correct content of which in the diet is of fundamental importance for the development of the organism, were found in all analyzed products. The presence of macroelements such as sodium, potassium, calcium, phosphorus, and magnesium, as well as microelements such as iron, zinc, copper, and manganese were confirmed in the tested milk.

Some analyzed products from HM group are manufactured for feeding in the first half of the year, hence they were included in FM group when comparing the obtained data with the tolerance range established in the European Union standards for baby food production. Similarly, one product from HM group dedicated for feeding in 6–12 month was included to OM milk group. Due to the period when material for research was collected and experiments were conducted (2019), the analysis used the EU Directive [27] in force until February 2021. The data were calculated in mg/100 kcal according to Directive guidelines and presented in Table 2.

The measured content of all essential elements, except P, was within the standard defined in the EU Directive regulating the quantitative and qualitative composition of first baby milk and follow-on milk (Table 2). In the case of P, the content of this element per 100 kcal significantly exceeded the acceptable standard, both in the FM and OM group. The values were 8.8 and 10.5 times higher, respectively.

### 2.2. Bioelements Content Discrepancies between Measured and Producent Declared Values

**Figure 2:**
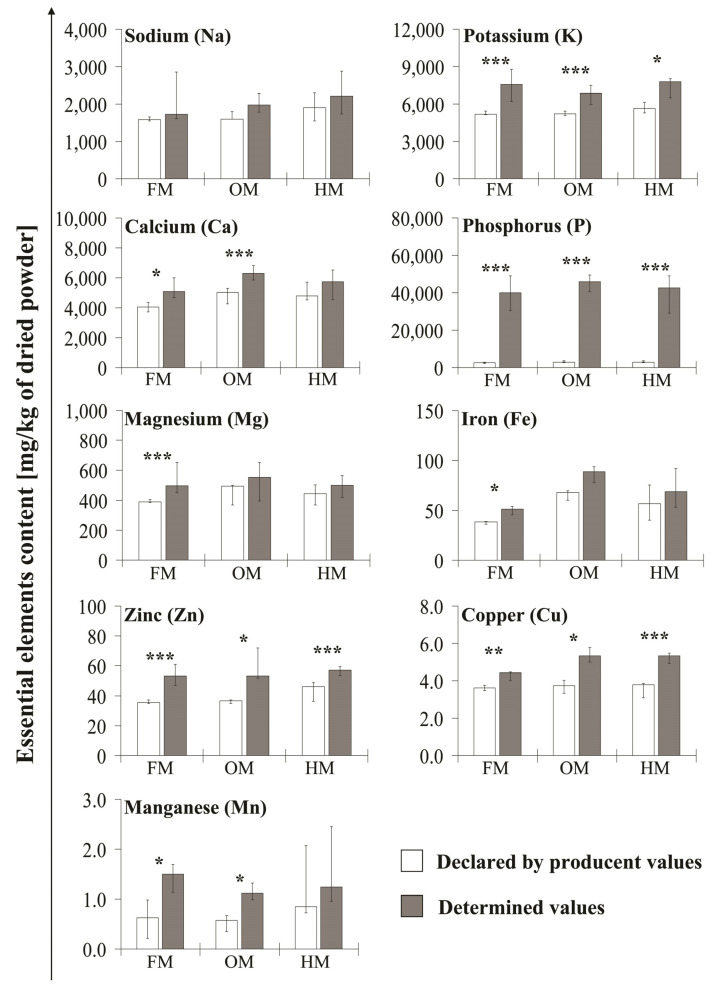
Composition discrepancies between obtained concentration values and declared by producers in analyzed baby milk. The comparison of obtained data with declared by producent content values presented on products labels. Data are presented as median (interquartile range) values of six experiments. FM—first baby milk, OM—follow-on milk, HM—hypoallergenic milk. The significance level of the differences observed: * *p* < 0.05; ** *p* < 0.01; *** *p* < 0.001.

Our analysis showed, in some cases, significant differences in the content of bioelements compared to the values declared by the producers. Composition discrepancies in first baby milk (FM), follow-on milk (OM), and hypoallergenic milk (HM) products are presented in Figure 2.

In the case of FM, the content of K, Ca, Mg, Fe, Zn, and Cu is slightly exceeded but with statistical significance the amount declared by the producer—the multiplicity of these values ranges from 1.3–1.6-times. Moreover, the measured content of P was dozen times higher than the declared concentration—on average 17-times with maximum in 30-times. Similarly, significantly higher content than the declared one was found for Mn—in range from 1.1–20.8-times. There were no statistically significant differences in Na content.

In the OM, the concentrations of K, Ca, Zn, and Cu were up to 1.4-times higher than those declared as statistically significant. As in the FM, the P content was much higher than the declared one—from 10 to 26.3-times. The determined content of Mn was higher, on average by 2.20-times. No statistically significant differences were found for Na, Mg, and Fe.

In HM, the concentrations of K, Zn, and Cu was slightly higher than the declared one but with statistical significance and ranges from 1.3–1.5-times, whereas for Na, Fe, and Mn no statistically significant differences were observed. In the case of P, the measured content of this element exceeded the declared value dozen times (10.1–17.2-times).

### 2.3. The Single Dose and Daily Administration of Essential Elements Contained in Analyzed Baby Milk

The frequency of feeding, and therefore also the daily intake of nutrients, depends on the age of a child and his health condition, but usually it is done "on demand". Procedures for preparing a solution of milk differ depending on the product. Based on the obtained measurement results and instructions on the preparation of the milk solution placed on the packaging labels, the dose of bioelements taken by the child fed with the tested products was estimated. Taking into account the data on the weight of dry powder in one spoon and the volume of water needed to prepare one food portion, the dose of bioelements in 100 mL of the final preparation was calculated. Data are presented in Table 3 for first baby milk (FM), follow-on milk (OM), and hypoallergenic milk for newborns (HM), respectively. The calculated amounts were compared with the recommended daily administration doses used for the Polish population [28] as well as with nutrition standards recommended by European Food Safety Authority (EFSA) [29].

**Figure 3:**
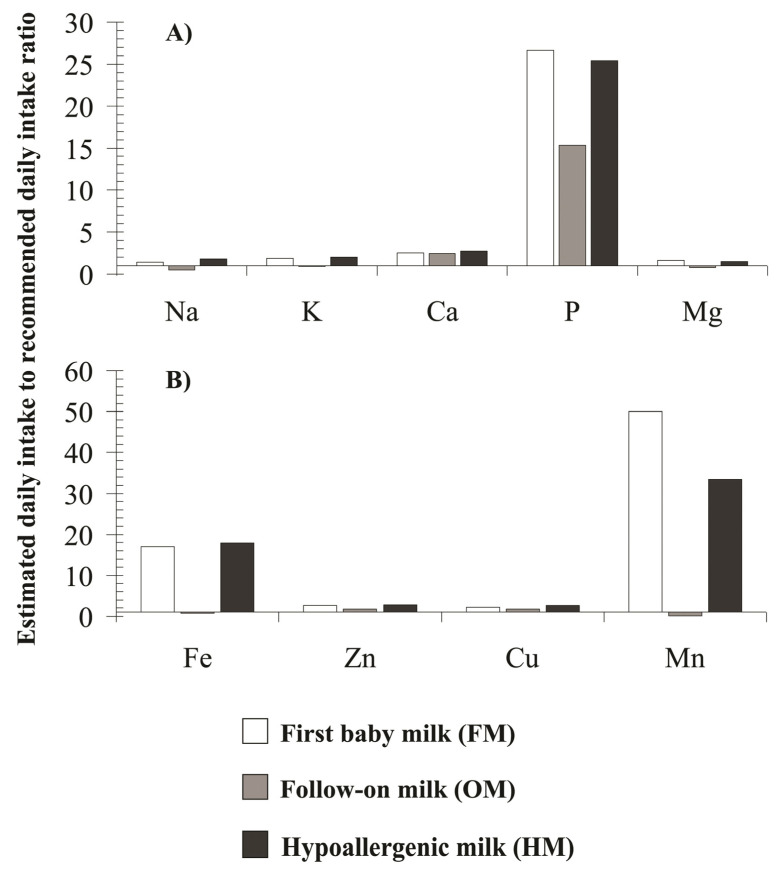
Coverage of the daily nutritional requirement for essential elements contained in analyzed baby milk calculated as estimated daily intake to recommended daily intake ratio. The daily supply of (**A**) macroelements, and (**B**) microelements contained in analyzed baby milk was estimated assuming that the minimum daily amount of the product (as a dry powder) that a child consumes is 100 g. The obtained values were compared to recommend for the Polish population.

Moreover, the coverage of the daily nutritional requirement for essential elements contained in the analyzed modified milk was estimated. Due to the fact that the feeding schedule is highly individualized, it was arbitrarily assumed that the minimum daily amount of the product that a child consumes is 100 g. The coverage of the daily nutritional requirement for essential elements contained in analyzed baby milk calculated as estimated daily intake to recommended daily intake ratio is presented in Figure 3A,B.

It was estimated that the daily supply of only P, Fe, and Mn, which is provided by nutrition with the tested products, significantly exceeds the recommended dose.

### 2.4. Contamination of Baby Milk with the Toxic Elements

**Figure 4:**
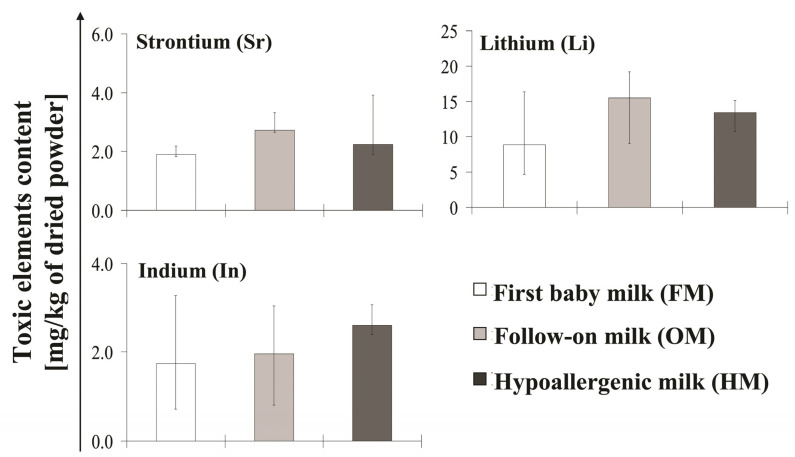
Concentration of the toxic elements contained in analyzed baby milk. The values of strontium (Sr), lithium (Li), and indium (In) content in analyzed first baby milk (FM), follow-on milk (OM), and hypoallergenic milk (HM). The data are presented as median (interquartile range) values of six determinations.

All types of tested baby milk were checked for presence of five toxic elements: Ni, Pb, Sr, Li, and In. Concentrations of harmful elements in each product analyzed are presented in Table 4 for first baby milk (FM), follow-on milk (OM), and hypoallergenic milk (HM), respectively. Data are given as a median value (with minimum and maximum level) for each analyzed product group. A comparison of the toxic elements content in individual milk groups is shown in Figure 4.

Among the tested toxic elements, only Ni was not present in any of the products. As for Pb, it was detected only in two products from the FM group (maximum concentration was 0.3 mg/kg), three products with OM group (with maximum 0.3 mg/kg) and in only one product of HM group (maximum 0.02 mg/kg).

In contrast, Sr and Li were present in all tested products. The concentrations of Sr were in range from 1.7–4.9 mg/kg, 2–3.4 mg/kg, and 1.5–4 mg/kg FM, OM, and HM group, respectively. In the case of Li the content was highest among all analyzed toxic elements. What is more, it varied from 2.9–23 mg/kg, 8.6–20.2 mg/kg, and 6.8–22.8 mg/kg in FM, OM, and HM group, respectively.

The presence of In was detected in all investigated products except one product from OM group. The concentration of In varied in range from 0.5–3.6 mg/kg, 0–3.9 mg/kg, and 2.3–4.4 mg/kg in FM, OM, and HM group, respectively.

## 3. Discussion

The content of the components in modified milk should meet the nutritional needs of children and ensure their proper growth and development [29].

Although opinions are divided, there is ample evidence that these products can be a source of dietary errors and may expose young children to environmental pollution and toxic metals [30,31,32,33,34].

Therefore, it is very important to study the content of ingredients in formula milk. In our study 18 modified baby milks available in Poland were analyzed. First baby milk (FM), follow-on milk (OM), and hypoallergenic milk (HM) with six different products in each group were analyzed qualitatively and quantitatively. They were tested in terms of the composition of the nine essential macro- and microelements, compliance with the declared composition by the manufacturer, and the presence of five toxic elements. An attempt to estimate the daily supply of elements using the tested products in relation to the recommended standards was also made.

The present paper demonstrates the level of essential elements, i.e., sodium, potassium, calcium, phosphorus, magnesium, iron, zinc, copper, and manganese presence in formula milks intended for feeding children in the first year of life. The presence of the required bioelements was confirmed in all tested products; however, their content varied in a fairly wide range dependently on the producer. The difference between the minimum and maximum content values measured in a given milk group was on average 2-times. The observed result is inconsistent with the data previously published by other authors, where the recorded values of Cu, Mn, Fe, and Zn content [35] or Cu and Zn content [36] between different baby milks available in Poland, as well as Fe, Mn, Zn, and Cu content in different products from Italy [37] or Cu and Zn content among formula from Austria [17] were similar. On the other hand, our data are consistent with the studies by Ljung et al. [5], where concentration of Ca, Fe, Mg, Zn, Cu, and Mn in artificial milk available in Sweden varied a little among examined products. In our study the highest content range was observed especially for Mn, where the difference was even 3.5-times and 6-times in FM and HM milk group, respectively. Similarly, high significant differences in mean values of Mn between products were also described by Lutfullah et al. [38] in formulas available in Pakistan and in mentioned above Jurowski et al. [36] and Ljung et al. [5] works.

The research also revealed discrepancies between the content declared by the producer and the measured value, especially in the case of K, P, Zn, and Cu, for which differences of high statistical significance were observed in the products from all tested milk groups. The content of Ca, Mg, Mn, and Fe in the FM milk group, as well as Ca and Mn in the hypoallergenic milk group, also did not match the data on the product labels. Similar differences in product composition were described by Jurowski et al. [36], where both lower and higher values than the declared concentrations were observed. The discrepancy between the labelled amount of the desired ingredients (energy substances, vitamins, or essential elements) and the actual state very often results from the so-called “overages”. Ingredients in food products and dietary supplements are added in larger amounts as their amount decreases during storage [22,39]. The products are therefore composed in such a way that at the end of the expiry date the content is not lower than the declared one. Nevertheless, while such production practices are accepted, discrepancies can lead to physiological and metabolic disturbances in the body. Moreover, it can also make it difficult to assess daily nutrient intake [11].

The content of macro- and microelements in modified milk is strictly defined and regulated in the EU countries by the Directive 2006/141/EC [27] (a new directive is now being introduced). Despite the observed significant discrepancies between different producers or between labelled and measured amounts, the content of almost all analyzed elements (8/9) was within the wide standards set by the EU Directive. The measurement concerned the production of modified milk, both for the initial and follow-on feeding of infants. The exception here was P, the content of which in all investigated products significantly exceeded the upper limit of the established norm. In the case of FM group, the amount was 9-times higher, and in milk from the OM group, it was as much as 11-times higher. Such discrepancy can seriously affect bone formation in formula-fed infants [40].

Although the tested products did meet the production standards in the case of most of essential elements, it should be noted that discrepancies in their nutritional composition may affect the daily amount of food biochemicals consumed, differing from the recommended standards [19,20,21]. In the presented study, an attempt was also made to assess the daily supply of elements provided by feeding a baby with the tested infant milk products. Basically, the issue of determining the level of absorption of nutrients and minerals in the youngest children is quite difficult due to the individualized development rate. Infants are fed "on demand" and their bodies are particularly exposed to higher doses of food substances because they eat more food per body weight and their gastrointestinal and renal systems are immature [37,41,42,43]. This is especially important for formula-fed children as modified milk contains higher levels of nutritional components than breast milk [5,15,42]. In 2013 EFSA established recommendation for nutrients requirements and dietary intakes of macro- and micronutrients for infants and young children [29]. The panel set the values of adequate intakes (AI) of bioelements based on mean intakes from breast milk per day. Polish nutritional standards are based on EFSA guidelines [28] and the values for most bioelements are convergent. In our study we calculated one-time administration dose (mg in 100 mL of final feed portion) and daily supply (mg in 100 g of consumed product) of bioelements contained in examined baby milk. In the case of Na, K, Ca, and Mg contained in FM group and HM for the first half of year, the daily average supply provides the daily requirement for these elements with a surplus of about 1.5–3-times. A much higher index was obtained for P. The daily dose consumed with milk from the FM group or HM group for the youngest children, is respectively 26- and 27-times higher than the nutritional norm and one-time administration portion of examined milk provides about 330–670 percent of adequate intake for P. The daily supply of Na, K, and Mg in milk from the OM group provides a lower amount of these macronutrients in relation to the AI value. It is desirable due to the solid food introduced during the follow-on milk feeding period and both types of food should complete the dietary needs. On the other hand, the child’s need for nutrients increases significantly in the second half of the first year of life, which is reflected in the higher values of nutritional standards [29,44]. The tested milks from the OM group provide about 2.5-times higher daily supply of Ca and as much as 15-times higher supply of P per day. The condition for proper skeletal development in children in the first year of life is to ensure an adequate supply of Ca and P [40]. Since the Ca to P ratio in breast milk is higher than in cow’s milk [45], children fed with modified cow’s based milk are at risk of hyperphosphatemia [46]. In the case of micronutrients, the daily supply of Zn and Cu provided in all tested products covers their respective consumption even up to 280 percent of recommended value. The adequate intake for Fe and Mn recommended for children 6–12 months of age is significantly higher [29] and daily supply with examined milk from OM group is insufficient. However, milk from FM group provides 17-times and 50-times more Fe and Mn, respectively, and from HM for early feeding, 18-times and 33-times more Fe and Mn than recommended adequate intake per day. High levels of Mn in modified milk supply per day were also noted in the studies of other authors. The studies of Chajduk et al. [35] revealed that estimated daily intakes of Fe and Mn in cow-based milk are 12-times higher than adequate values. In Ljung et al. work [5] the calculated daily intake of Fe and Mn was 44- and 190-times, respectively higher than amount provided with maternal milk. Estimated daily intakes of Fe and Mn significantly exceeding the recommended values (18-times and 23–51-times, respectively) are also reported by Bargellini et al. [37]. Similar results were obtained in a study of Spanish and Serbian infant milk when the increased mean daily intake of Fe and Mn was also calculated [47]. As in our results, the daily intake of Mn with modified milk exceeding recommended value was also reported by Krachelr and Rossipal [17], Sipahi et al. [42], and Pandelova et al. [41]. The increased content of bioelements in the formula milk may affect the overall supply and absorption level of this element in gastrointestinal track. In the case of Mn, it is particularly important in the context of the potential neurotoxicity of this element. Infants are sensitive to Mn exposure due to the higher absorption and low elimination rate of Mn [48]. It is confirmed that the long-term exposure to Mn induces neurobehavioral disorders in children [49]. Likewise, improper Fe intake in children can cause numerous disorders. Deficiencies cause poor development of the nervous system and anemia, while overload disrupts iron absorption homeostasis and leads to oxidative stress and a weakened immune system [50].

Toxic contaminants in food for children are of great concern. A potential source of artificial milk contamination is the raw material derived from diary animals, manufacturing process, packaging materials, improper storage, and preparation of food portions. Such contaminants include pesticides, non-essential toxic elements, drugs, mycotoxins, chemical compounds of heat treatments, or persistent organic pollutants [51]. Since infants are susceptible to heavy metal toxicity due to rapid growth and development of organs and tissues, especially central nervous system, so far milk for newborns and infants has been tested for the presence of toxic elements such as: Cd, As, Hg, Be, Ti, Sn, Tl, U, V, Sb, Ba, La, Li, and Rb, as well as Ni, Pb, and Sr, which were the subject of research in this paper [5,14,15,17,37,41,42,52,53,54,55]. 

Although there is evidence that the modified milk may contain Ni, our research did not reveal the presence of this element in the tested products. Our data are inconsistent with Chajduk et al. study [35], where Ni was found in breast milk substitutes in range 70.9–586 ng/g as well as in cereal also available in Poland. One of the most common toxic food contaminants is Pb, the neurotoxic effect of which is well known [56]. In the tested products Pb was found only in six products (2/6 in FM group, 3/6 in OM group, and 1/6 in HM group) and the content value exceeded permissible limit of 0.05 mg/kg established in EU Commission Regulation (2006) [57] in the three of them in range of 2.6–6.8-times. Higher than the established in our study Pb content in modified milk was found in other authors [52,58]. In many cases estimated daily Pb exposure was within the safety limits [13,32,35,38,41,42,53,55].

There is a very little data concerning Sr and Li in infant formula. According to our results, the whole tested formula was contaminated with these elements. The presence of Sr and Li in infant formula was confirmed by Krachel and Rossipal [17] and Krachler et al. [54] teams. However, determined Sr and Li concentrations in the first formula, follow-on formula and extensively hydrolyzed formula where on much lower level. The content of Sr in some infant formula from USA, UK and Nigeria was also confirmed [53]. According to FSA data available on Sr and Li are limited and did not allow a full assessment of its safety and toxicity toward children [18]. The exposure of infants to Li could result from a transfer from an exposed mother (due medication) [59]. However, according to Tamari [60], the presence of Li in formulas could be the impurity from manufacturing process.

For the first time in the literature on the subject, we show the presence of In in modified milk. This trace element is used for the production of semiconductors, solders, and bearings, whereas in medicine for scanning and cancer treatment. Humans have been shown to absorb In from the milk of animals grazed around industrial areas [18]. It was also reported as a cause of disorders of inflammatory factors [61].

Furthermore, it should be mentioned, that the level of the daily intake and absorption of the bioelements both essential and toxic taken with the modified milk is also influenced by the quality of the water, which is used to dissolve powdered preparation [54,62].

## 4. Materials and Methods

### 4.1. Samples Characteristic

Eighteen products, i.e., six first baby milk (FM; for newborns and up to 6 months of feeding), six follow-on milk (OM; for feeding from 6 to 12 months), and six hypoallergenic milk (HM; five products for newborns and one product for infants) from different producers based on cow-milk or protein hydrolysates were analyzed. All products were manufactured in European countries and available for sale in markets and pharmacies in Poland. Individual products have been marked as: FM-1–FM-6, OM-1–OM-6, and HM-1–HM-6 for first baby milk, follow-on milk, and hypoallergenic milk, respectively.

### 4.2. Samples Preparation and Chemical Analysis

**Figure 5:**
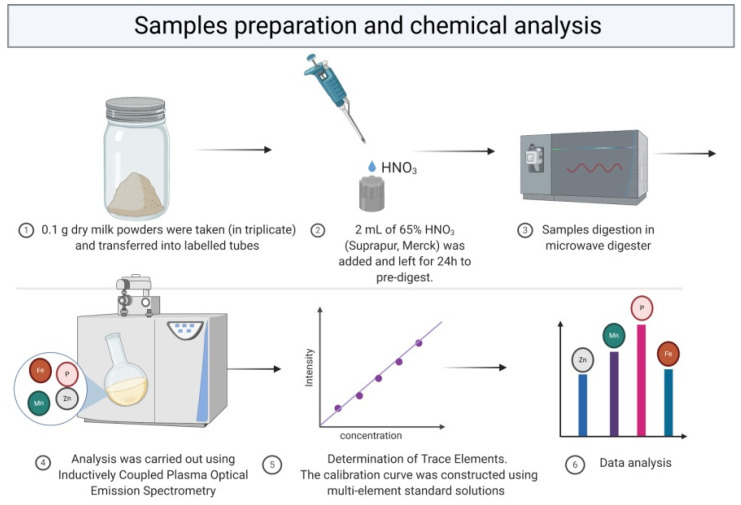
The workflow of samples preparation and chemical analysis (Created with BioRender.com).

The collected samples were pre-processed and then analyzed according to the scheme described below (Figure 5).

#### 4.2.1. Samples Pre-Treatment and Mineralization

For the purposes of the study, samples of 0.1 g dry milk powders were taken (in triplicate) and transferred into labelled tubes. Then, 2 mL of 65% HNO_3_ (Suprapur, Merck) was added and left for 24 h to pre-digest. The samples were then transferred to Teflon vessels and placed in the microwave digester, where the digestion process took place in two stages. During stage one (15 min), the samples were gradually heated up to 180 °C, and during stage two (20 min) the temperature was maintained at 180 °C. At the end of the decomposition process, the samples were transferred into new tubes.

#### 4.2.2. Determination of Elements

The digested samples were diluted 20-times and then an internal standard, 500 µL of yttrium, was added at final concentration of 0.5 mg/L and 1 mL of 1% Triton (Triton X-100, Sigma). The samples were diluted with 0.075% HNO_3_ (Suprapur, Merck) up to the volume of 10 mL, and stored in the fridge until analysis.

Bioelements (Na, K, Ca, P, Mg, Fe, Zn, Cu, and Mn) and toxic elements (Ni, Pb, Sr, Li, and In) analysis was carried out using inductively coupled plasma optical emission spectrometry (ICP-OES, ICAP 7400 Duo, Thermo Scientific) equipped with a concentric nebulizer and a cyclonic spray chamber. Analyzed elements have detection limits of a few part per billion (ppb, ng/mL).

### 4.3. Analytical Calibration and Quality Control

The calibration curve was constructed using multi-element standard solutions (ICP multi-element standard solution IV, IX, and XVI, Merck; 100 mg/L of each element), which were prepared with deionized water (Direct Q UV, Millipore, approx. 18.0 MΩ). Multi-element calibration standards were prepared with different concentrations of inorganic elements in the same manner as in blanks and samples. Samples of reference material (NIST SRM 1486 Bone Meal) (n = 3) were prepared in the same manner as samples (Table 5). The blank test was prepared according to the same procedure, with the study sample replaced by 250 µL of nitric acid (V).

### 4.4. Statistical Analysis

All statistical analyses were performed using Statistica ver.13 software. The normality of the data distribution was verified in the Shapiro–Wilk test. Statistical analysis of the significance level of differences observed between analyzed groups was carried out with nonparametric Mann–Whitney U test. *p* value less than 0.05 was considered as a significant difference.

## 5. Conclusions

Discrepancies between the values declared by the manufacturer and the actual concentration values of the ingredients of the preparation (even resulting from “overages”) could influence the daily intake of macro- and micronutrients. Formula feeding could increase the body’s exposure to toxic elements inducing severe implications in child’s health. There is a need to regularly monitor the quantitative and qualitative composition of formula milk in order to ensure the safety of food for children. It ought to be done both in the context of the correct supply of essential elements defined in the nutritional standards, and the exposure to toxic metal in food. Presented study can be useful in further studies on baby food safety and health risk assessment.

## Figures and Tables

**Table 1:** The essential elements content in analyzed baby milks (mg/kg of dried powder).

	Product Group								
Essential Elements	First Baby Milk (FM)			Follow-on Milk (OM)			Hypoallergenic Milk (HM)		
	Determined Content [mg/kg of Dried Powder]								
	Median	Min	Max	Median	Min	Max	Median	Min	Max
Sodium	1730	1535	3220	1981	1382	2459	2212	1617	3066
Potassium	7588	6058	9425	6861	5953	9399	7790	4896	12,246
Calcium	5075	3884	7180	6309	5763	7240	5744	3939	7183
Phosphorus	39,952	24,960	80,796	45,963	34,857	70,025	42,683	26,237	67,361
Magnesium	496	441	1203	553	363	1207	500	406	628
Iron	51	28	54	89	48	100	69	45	109
Zinc	53	43	67	53	51	82	57	51	61
Copper	2	4	6	5	3	6	5	4	6
Manganese	2	1	5	1	1	2	1	1	3

**Table 2:** The comparison of obtained data with tolerance range established in the European Union standards according to EU Commission Directive 2006/141/EC (2006) [27].

Essential Elements	Tolerance Range According to the UE Commission Directive 2006/141/EC (2006) [mg/100 kcal]	Determined Content Calculated to mg/100 kcal (Median)
	**First Baby Milk Group (FM) and** **Hypoallergenic Milk (HM) for the First Half of Year**	
Sodium	20–60	35
Potassium	60–160	159
Calcium	50–140	102
Phosphorus	25–90	793
Magnesium	5–15	9
Iron	0.3–1.3	1.1
Zinc	0.5–1.5	1.1
Copper	0.035–0.1	0.09
Manganese	0.001–0.1	0.03
	**Follow-on Milk Group (OM) and** **Hypoallergenic Milk (HM) for the Second Half of Year**	
Sodium	20–60	42
Potassium	60–160	127
Calcium	50–140	127
Phosphorus	25–90	944
Magnesium	5–15	11
Iron	0.6–2	1.7
Zinc	0.5–1.5	1.1
Copper	0.035–0.1	0.11
Manganese	0.001–0.1	0.03

**Table 3:** The administration dose of the essential elements contained in a single portion of final preparation (mg/100 mL) in regard to the nutritional standards for the Polish population [28] and European Food Safety Authority (EFSA) recommendations [29]. Additional information on the qualitative and quantitative composition of all ingredients of the tested products, as declared by the manufacturer, is provided in Table S1(a–c) in the Supplemental Data.

Elements	Standards Established for the Polish Population [mg/day]	Nutrition Standards Recommended by EFSA (2013) [mg/day]	The Determined Content Calculated to mg in 100 mL of the Final Preparation of the Analyzed Products		
			Median	Min	Max
	**First baby milk (FM)**				
Sodium	120 *	120 *	22	21	48
Potassium	400 *	400 *	105	78	141
Calcium	200 *	200 *	68	50	108
Phosphorus	150 *	100 *	551	322	1212
Magnesium	30 *	25 *	7	6	18
Iron	0.3 *	0.3 *	0.7	0.4	0.7
Zinc	2 *	2 *	0.7	0.6	1
Copper	0.2 *	0.3 *	0.1	0.1	0.1
Manganese	0.003 *	0.003 *	0.02	0.01	0.06
	**Follow-on milk (OM)**				
Sodium	370 *	170–370 *	28	20	37
Potassium	750 *	800 *	93	86	141
Calcium	260 *	400 *	88	81	101
Phosphorus	300 *	300 *	640	502	1050
Magnesium	70 *	80 *	8	5	18
Iron	11 **	8 *	1.2	0.7	1.4
Zinc	3 **	4 *	0.8	0.7	1.1
Copper	0.3 *	0.3 *	0.1	0.04	0.1
Manganese	0.6 *	0.02–0.5 *	0.02	0.01	0.03
	**Hypoallergenic milk (HM) for the first half of year**				
Sodium	120 *	120 *	29	22	41
Potassium	400 *	400 *	104	88	165
Calcium	200 *	200 *	70	53	97
Phosphorus	150 *	100 *	492	354	909
Magnesium	30 *	25 *	6	6	8
Iron	0.3 *	0.3 *	0.7	0.6	1.5
Zinc	2 *	2 *	0.8	0.7	0.8
Copper	0.2 *	0.3 *	0.1	0.1	0.1
Manganese	0.003 *	0.003 *	0.01	0.01	0.04

* Adequate Intake (AI); ** Recommended Dietary Allowance (RDA).

**Table 4:** The toxic elements content in analyzed baby milk (mg/kg of dried powder).

Essential Elements	Product Group								
	First Baby Milk (FM)			Follow-on Milk (OM)			Hypoallergenic Milk (HM)		
	Determined Content [mg/kg of Dried Powder]								
	Median	Min	Max	Median	Min	Max	Median	Min	Max
Nickel	n.d.	-	-	n.d.	-	-	n.d.	-	-
Lead	0	0	0.3	0.01	0	0.3	0	0	0.02
Strontium	1.9	1.7	4.9	2.7	2	3.4	2.3	1.5	4
Lithium	8.9	2.9	23	15.5	8.6	20.2	13.4	6.8	22.8
Indium	1.7	0.5	3.6	2	0	3.9	2.6	2.3	4.4

n.d.—not detected.

**Table 5:** Analysis of reference material Bone Meal NIST-SRM 1486 using ICP-OES.

Chemical Element	NIST-SRM 1486 Certified [mg/kg]	NIST-SRM 1486 Measured [mg/kg]	Recovery (%)
P 178.284	123,000	131,500	107%
K 766.490	412	417	101%
Ca 315.887	265,800	247,021	93%
Fe 239.562	99	102.15	103%
Zn 213.856	147	143.60	98%
Sr 421.552	264	258.17	98%
Na 589.592	5000	4826.33	97%
Mg 285.213	4600	4382.13	95%
Cu 324.754	0.80	0.78	97.5%
Mn 403.076	1.00	0.97	97%
Pb 283.310	1.335	1.307	98%

## Data Availability

The data declared by the producers concerning the qualitative and quantitative composition of the ingredients were taken from the labels on the packaging of individual products.

## Supplementary Materials

The following are available online. Table S1(a): The content of the ingredients in first baby milk analyzed declared by the manufacturers. Table S1(b): The content of the ingredients in follow-on baby milk analyzed declared by the manufacturers. Table S1(c): The content of the ingredients in hypoallergenic baby milk analyzed declared by the manufacturers.

## Sample Availability

Samples of the compounds are not available from the authors.

## Abbreviations

AI—Adequate Intake; FM—first baby milk, HM—hypoallergenic milk, ICP OES—Inductively Coupled Plasma Optical Emission Spectrometry; OM—follow-on milk; RDA—Recommended Dietary Allowance.
