# Supplementary material for: Modified Baby Milk—Bioelements Composition and Toxic Elements Contamination
Source: Molecules. 2021 Jul 9;26(14):4184. doi: 10.3390/molecules26144184 (PMC8307874; doi:10.3390/molecules26144184)
Supplement: Supplementary file 1 [file molecules-26-04184-s001.zip › molecules-1289207-supplementary.pdf]

molecules

Supplemental Data

Table S1a. The content of the ingredients in first baby milk analyzed declared by the manufacturers.

Ingredient	Unit	FM-1	FM-2	FM-3	FM-4	FM-5	FM-6
		Product code declared value in 100 mL of final preparation					
**Energy**	**kcal**	66	66	66	67	68	70
**Fat (total)**	**g**	3.4	3.4	3.5	3.56	3.6	4
**Saturated fatty acids**	**g**	1.5	1.5	1.2	0.84	1.3	n.d.
**Monounsaturated fatty acids**	**g**	1.3	1.3	1.6	1.78	n.d.	n.d.
**Polyunsaturated fatty acids (total)**	**g**	0.6	0.6	0.7	0.63	n.d.	n.d.
**Linoleic acid (LA)**	**mg**	448	446	n.d.	555	n.d.	n.d.
**α-Linolenic acid (ALA)**	**mg**	55	54.3	n.d.	49	n.d.	n.d.
**Arachidonic acid (ARA)**	**mg**	16.5	16.5	12	n.d.	n.d.	n.d.
**Docosahexaenoic acid (DHA)**	**mg**	16.5	16.5	7	17.4	n.d.	n.d.
**Eicosapentaenoic acid (EPA)**	**mg**	3.6	3.5	n.d.	n.d.	n.d.	n.d.
**Carbohydrates (total)**	**g**	7.4	7.4	7.3	7.45	7.4	8.4
**Lactose**	**g**	7	7	n.d.	7.245	5.7	n.d.
**Dietary fiber**	**g**	0.6	0.6	0.3	0.1	n.d.	0.47
**Galactooligosaccharides**	**g**	0.48	0.48	0.3	n.d.	n.d.	n.d.
**Fructooligosaccharide**	**g**	0.08	0.08	n.d.	n.d.	n.d.	n.d.
**3-Galactosyllactose**	**g**	n.d.	0.015	n.d.	n.d.	n.d.	n.d.
**Proteins**	**g**	1.3	1.3	1.25	1.24	1.4	1.6
**L-carnitine**	**mg**	2.1	2.1	n.d.	1.23	n.d.	n.d.
**Vitamin A**	**µg**	58	58	70	60.65	58	62
**Vitamin D**	**µg**	1.6	1.4	1.2	1.67	1.2	1.6
**Vitamin E**	**mg**	1.2	1.1	0.9	1.33	1.8	1.3
**Vitamin K**	**µg**	4.4	5.8	5	4.52	7.7	4.7
**Vitamin C**	**mg**	9.2	9.2	10	10.33	11	10
**Thiamine**	**µg**	60	60	60	65	70	48
**Riboflavin**	**µg**	140	140	100	155	200	124
**Niacin**	**mg**	0.43	0.43	0.4	0.52	0.6	0.5
**Vitmin B6**	**µg**	48	48	40	39	50	40
**Folic acid**	**µg**	8	8.2	10	19.36	24	13
**Vitamin B12**	**µg**	0.09	0.17	0.15	0.17	0.2	0.2
**Biotin**	**µg**	1.8	1.7	1.5	1.42	2.4	1.4
**Pantothenic acid**	**mg**	0.53	0.53	0.5	0.54	0.7	0.34
**Sodium**	**mg**	21.9	21.9	20	21.3	30	20
**Potassium**	**mg**	71	71	70	67.11	91	75
**Chloride**	**mg**	52	52	45	50.33	51	48
**Calcium**	**mg**	61	60	50	42.59	56	56
**Phosphorus**	**mg**	42	41	27	23.879	40	40
**Magnesium**	**mg**	5.4	5.4	5	5.68	6.6	5.4
**Iron**	**mg**	0.53	0.53	0.5	0.31	0.6	0.54
**Zinc**	**mg**	0.48	0.48	0.5	0.48	0.6	0.54
**Copper**	**µg**	52	52	45	54	40	42
**Manganese**	**µg**	3	3	7.5	20.65	11	10
**Fluoride**	**µg**	≤6	≤6	≤5	≤7.74	5	3
**Selenium**	**µg**	3	3	1.3	3.87	2.3	1.7

**Iodine**	**µg**	13	13	15	14.2	15	12
**Molybdenum**	**µg**	n.d.	n.d.	n.d.	n.d.	n.d.	n.d.
**Chromium**	**µg**	n.d.	n.d.	n.d.	n.d.	n.d.	n.d.
**Inositol**	**mg**	7	7.2	3.9	9.03	5.3	4.8
**Taurine**	**mg**	5.3	n.d.	n.d.	4.13	n.d.	n.d.
**Choline**	**mg**	22	22	11	21.94	12	12
**Nucleotides**	**mg**	2.3	2.3	n.d.	1.91	n.d.	n.d.
**Immunoglobulins**	**mg**	n.d.	n.d.	n.d.	n.d.	n.d.	2.3
**Probiotics bacteria**	**-**	n.d.	n.d.	included	included	n.d.	n.d.

n.d. – no data

Table S1b. The content of the ingredients in follow-on baby milk analyzed declared by the manufacturers.

Ingredient	Unit	Product code OM-1	OM-2	OM-3	OM-4	OM-5	OM-6
		declared value in 100 mL of final preparation					
**Energy**	**kcal**	68	68	68	67	65	68
**Fat (total)**	**g**	3.2	3.2	3.7	3.2	2.9	3.42
**Saturated fatty acids**	**g**	1.4	1.4	1.6	0.8	n.d.	1.8
**Monounsaturated fatty acids**	**g**	1.2	1.2	1.6	1.6	n.d.	0.9
**Polyunsaturated fatty acids (total)**	**g**	0.5	0.5	0.5	0.6	n.d.	0.72
**Linoleic acid (LA)**	**mg**	425	428	420	469	n.d.	650
**α-Linolenic acid (ALA)**	**mg**	52.2	51.4	60	45.6	n.d.	70
**Arachidonic acid (ARA)**	**mg**	1.2	8.8	n.d.	n.d.	n.d.	n.d.
**Docosahexaenoic acid (DHA)**	**mg**	17	17	13.6	16.8	n.d.	n.d.
**Eicosapentaenoic acid (EPA)**	**mg**	3.6	3.6	n.d.	n.d.	n.d.	n.d.
**Carbohydrates (total)**	**g**	8.2	8.2	7.2	8.4	7.5	7.6
**Lactose**	**g**	7.2	7.8	7	8.4	n.d.	n.d.
**Dietary fiber**	**g**	0.6	0.6	0.4	0.026	1.25	n.d.
**Galactooligosaccharides**	**g**	0.48	0.48	0.4	n.d.	n.d.	0.360
**Fructooligosaccharide**	**g**	0.08	0.08	n.d.	n.d.	n.d.	0.04
**3-Galactosyllactose**	**g**	n.d.	0.015	n.d.	n.d.	n.d.	n.d.
**Proteins**	**g**	1.4	1.4	1.3	1.1	2.3	1.6
**L-Carnitine**	**mg**	n.d.	n.d.	n.d.	n.d.	n.d.	1.1
**Vitamin A**	**µg**	60	60	56	61.6	69	69
**Vitamin D**	**µg**	1.7	1.7	1.6	1.7	1.5	1.1
**Vitamin E**	**mg**	0.86	0.77	0.8	1.4	1.4	1.1
**Vitamin K**	**µg**	4.5	5.9	3.1	5.1	5	4
**Vitamin C**	**mg**	8.8	9	10	9.6	10	11
**Thiamine**	**µg**	50	50	37	70	49	60
**Riboflavin**	**µg**	140	140	140	150	122	180
**Niacin**	**mg**	0.46	0.46	0.53	0.5	0.5	0.63
**Vitamin B6**	**µg**	46	45	42	40	40	80
**Folic acid**	**µg**	7.7	8	16.6	11.3	17	13
**Vitamin B12**	**µg**	0.09	0.17	0.1	0.16	0.18	0.25
**Biotin**	**µg**	1.8	1.8	1.6	1.7	1.5	2.8
**Pantothenic acid**	**mg**	0.48	0.49	0.35	0.4	0.34	0.5
**Sodium**	**mg**	23	23	20	34.2	24	25
**Potassium**	**mg**	75	78	84	91.1	73	70
**Chloride**	**mg**	52	54	52	48.2	48	41
**Calcium**	**mg**	72	73	70	65.7	64	73
**Phosphorus**	**mg**	50	50	40	40.2	40	39
**Magnesium**	**mg**	7.1	7.1	6.3	4.4	5.4	7
**Iron**	**mg**	1	1	1	0.9	1	1.1
**Zinc**	**mg**	0.5	0.5	0.5	0.5	0.54	0.9
**Copper**	**µg**	54	54	54	60	42	60
**Manganese**	**µg**	5	5	7	20	10	8
**Fluoride**	**µg**	≤6.1	≤6	≤10	≤10	3	≤70
**Selenium**	**µg**	3.1	3.1	3.9	3.4	1.7	1.5
**Iodine**	**µg**	13	13	15	14.1	12	10
**Molybdenum**	**µg**	n.d.	n.d.	n.d.	n.d.	n.d.	n.d.

**Chromium**	**µg**	n.d.	n.d.	n.d.	n.d.	n.d.	n.d.
**Inositol**	**mg**	n.d.	6.6	n.d.	n.d.	4.7	3.3
**Taurine**	**mg**	5.1	n.d.	n.d.	n.d.	n.d.	4
**Choline**	**mg**	n.d.	15	n.d.	n.d.	12	9.6
**Nucleotides**	**mg**	2.4	2.4	n.d.	n.d.	n.d.	3.18
**Immunoglobulins**	**mg**	n.d.	n.d.	n.d.	n.d.	n.d.	n.d.
**Probiotics bacteria**	**-**	n.d.	n.d.	included	included	n.d.	n.d.

n.d. – no data

Table S1c. The content of the ingredients in hypoallergenic baby milk analyzed declared by the manufacturers.

Ingredient	Unit	Product code					
		HM-1	HM-2	HM-3	HM-4	HM-5	HM-6
		declared value in 100 mL of final preparation					
**Energy**	**kcal**	68	66	65	66	67	67
**Fat (total)**	**g**	3.4	3.4	3.2	3.4	3.08	3.3
**Saturated fatty acids**	**g**	1.46	1.6	n.d.	1.6	0.85	1.2
**Monounsaturated fatty acids**	**g**	n.d.	1.2	n.d.	1.2	1.44	1.4
**Polyunsaturated fatty acids (total)**	**g**	n.d.	0.5	n.d.	0.6	0.56	0.7
**Linoleic acid (LA)**	**mg**	610	n.d.	562	448	479	n.d.
**α-Linolenic acid (ALA)**	**mg**	46	n.d.	82	54.3	60	n.d.
**Arachidonic acid (ARA)**	**mg**	23	n.d.	6.4	16.5	n.d.	n.d.
**Docosahexaenoic acid (DHA)**	**mg**	11.6	6.4	6.4	16.5	n.d.	n.d.
**Eicosapentaenoic acid (EPA)**	**mg**	n.d.	n.d.	n.d.	3.6	n.d.	n.d.
**Carbohydrates (total)**	**g**	7.5	7.2	7.6	7.1	8.54	7.8
**Lactose**	**g**	0	n.d.	5.5	2.9	n.d.	0
**Dietary fiber**	**g**	n.d.	0.06	0	0.5	n.d.	n.d.
**Galactooligosaccharides**	**g**	n.d.	0.8	n.d.	0.47	n.d.	n.d.
**Fructooligosaccharide**	**g**	n.d.		0	0.08	n.d.	n.d.
**3-Galactosyllactose**	**g**	n.d.	n.d.	0	n.d.	n.d.	n.d.
**Proteins**	**g**	1.91	1.5	1.5	1.6	1.27	1.7
**L-Carnitine**	**mg**	1.7	n.d.	1.6	2.1	n.d.	1.6
**Vitamin A**	**µg**	61	50	66	58	69.74	67
**Vitamin D**	**µg**	1.03	1.2	1.1	1.7	0.93	0.95
**Vitamin E**	**mg**	0.91	1	0.9	1.2	1.23	0.77
**Vitamin K**	**µg**	8.9	4.4	5.5	4.4	5.06	5.9
**Vitamin C**	**mg**	14.3	9.1	11	9.1	10.94	8.4
**Thiamine**	**µg**	55	50	65	70	70	61
**Riboflavin**	**µg**	61	100	98	140	150	102
**Niacin**	**mg**	0.68	0.43	0.592	0.87	0.73	0.676
**Vitamin B6**	**µg**	41	40	62	45	50	63
**Folic acid**	**µg**	10.9	8.4	11	8.7	11.08	8.4
**Vitamin B12**	**µg**	0.2	0.16	0.16	0.16	0.17	0.14
**Biotin**	**µg**	2	1.8	2	1.9	1.5	8.4
**Pantothenic acid**	**mg**	0.34	0.35	0.359	0.57	0.69	0.439
**Sodium**	**mg**	32	25	20	20.8	30.08	27
**Potassium**	**mg**	83	73	72	87	57.43	77
**Chloride**	**mg**	65	41	44	51	50.59	47
**Calcium**	**mg**	77	46	59	61	77.12	68
**Phosphorus**	**mg**	53	26	33	35	46.9	40
**Magnesium**	**mg**	6.8	5.1	5.1	5	6.84	6.6
**Iron**	**mg**	1.23	0.53	0.6	0.54	0.99	0.9
**Zinc**	**mg**	0.48	0.5	0.6	0.66	0.6	0.7
**Copper**	**µg**	51	40	40	52	50	53
**Manganese**	**µg**	41	10	12	8	10	28
**Fluoride**	**µg**	n.d.	≤10	12	≤6	≤8	31
**Selenium**	**µg**	1.5	1.5	2	3	2.6	1.4
**Iodine**	**µg**	14.3	12	11	13	9.57	8.6
**Molybdenum**	**µg**	3.8	n.d.	n.d.	5.9	n.d.	3

**Chromium**	**µg**	1.5	n.d.	n.d.	5.9	n.d.	1.8
**Inositol**	**mg**	11.6	4.1	5.2	n.d.	n.d.	6.3
**Taurine**	**mg**	4.1	n.d.	4.6	5.3	n.d.	3.8
**Choline**	**mg**	16.4	10	20	22	n.d.	8.4
**Nucleotides**	**mg**	n.d.	3.2	n.d.	2.3	n.d.	n.d.
**Immunoglobulins**	**mg**	n.d.	n.d.	n.d.	n.d.	n.d.	n.d.
**Probiotics bacteria**	**-**	included	n.d.	n.d.	n.d.	included	n.d.

n.d. – no data
